# LL‐37 IgG levels are associated with clinical characteristics and T follicular cell response in acute coronary syndrome in adults

**DOI:** 10.14814/phy2.70914

**Published:** 2026-06-16

**Authors:** Paul C. Dimayuga, Jianchang Zhou, Xiaoning Zhao, Kuang‐Yuh Chyu, Prediman K. Shah, Bojan Cercek

**Affiliations:** ^1^ Oppenheimer Atherosclerosis Research Center, Department of Cardiology Smidt Heart Institute, Cedars‐Sinai Medical Center Los Angeles California USA

**Keywords:** acute coronary syndrome, autoantibody, cathelicidin, LL‐37, T follicular cells

## Abstract

Elevated LL‐37 IgG levels are implicated in inflammatory conditions and immune complexes formed with LL‐37 potentially propagate immunothrombosis in acute coronary syndrome (ACS). The reported binding of LL‐37 to LDL may be relevant in this context given that LDL autoantibodies are implicated in ACS risk. In this report, three distinct cohorts were used to evaluate immunologic characteristics, clinical relevance, and potential mechanisms of elevated LL‐37 IgG levels in ACS. First, evaluation of IgG reactive to native (n) LDL and LL‐37 complexed with LDL (LL‐37_LDL) in a cohort without acute disease demonstrated significantly higher levels of LL‐37 IgG compared to LL‐37_LDL IgG and nLDL IgG. Next, evaluation of LL‐37 IgG levels in samples (*N* = 500) from the AZACS study (Azithromycin in ACS) and population‐level associations with clinical characteristics showed that LL‐37 IgG levels were significantly higher in subjects who had prior myocardial infarction and were negatively correlated with blood pressure. Third, an ACS cohort showed a negative association of LL‐37 IgG with LVEF. There was also significantly reduced CD8^+^CD25^+^CXCR5^+^ T follicular (Tf) regulatory cells with constant CD8^+^CD137^+^CXCR5^+^ Tf‐like cells in response to LL‐37 stimulation of peripheral blood mononuclear cells suggesting the association of Tf cells with increased LL‐37 IgG levels in ACS.

## 1 INTRODUCTION

LL‐37 is the only known cathelicidin‐type antimicrobial peptide in humans and it promotes inflammatory signaling in various disease pathologies (Kahlenberg & Kaplan, 2013). It is a component of neutrophil extracellular traps (NETs) (Bosch, 2011; Lande et al., 2011) implicated in thrombus formation (Pircher et al., 2018). Increased LL‐37 mRNA expression in peripheral blood mononuclear cells (PBMCs) was associated with early‐onset ST‐segment elevation myocardial infarction (STEMI) (Szauter et al., 2011). LL‐37 expression was also associated with risk factors for atherosclerotic cardiovascular disease (ASCVD) (Benachour et al., 2009). In patients with coronary artery disease (CAD), LL‐37 plasma levels had a positive association with blood pressure and triglyceride levels, while a negative association was reported for left ventricular ejection fraction (LVEF) in an acute coronary syndrome (ACS) subcohort (Höpfinger et al., 2024). Conversely, a different study reported that LL‐37 levels were reduced in STEMI patients and lower systemic LL‐37 levels were associated with a higher risk of a recurrent event (Zhao et al., 2012, 2022). Thus, although evidence suggests a link between LL‐37 and acute coronary events, the physiologic role of the peptide itself is unclear.

To investigate the nature of these contradictions, we looked beyond the peptide levels and instead examined the host immune response to LL‐37 (Chernomordik et al., 2023; Dimayuga et al., 2024). In autoimmune diseases, the pathogenic role attributed to LL‐37 involves a feed‐forward mechanism where inflammatory signaling promotes neutrophil discharge of NETs which bind pre‐existing LL‐37 autoantibodies (Bosch, 2011). These immune complexes (ICs) formed by LL‐37 and LL‐37 IgG autoantibodies then bind Fc gamma receptor 2a (FcɣRIIa) expressed by platelets and neutrophils which amplify the inflammatory response (Bosch, 2011) and potentially propagate immunothrombosis. Our studies suggested that this is a potentially relevant pathway for LL‐37 involvement in acute coronary events. We have reported that, in a study evaluating ASCVD risk using coronary artery calcium scans, subjects who experienced an acute myocardial infarction (MI) during a 13‐year follow‐up (Future MI group) had significantly higher baseline LL‐37 IgG levels compared to those who did not (Dimayuga et al., 2024). This Future MI group also had significantly higher LDL levels and CAC score, both known risks for an acute coronary event. Plasma from patients admitted for ACS had significantly increased levels of LL‐37 IgG‐ICs that promoted platelet activation mediated in part by FcɣRIIa (Dimayuga et al., 2024). We have also demonstrated that T cells in ACS patients lack tolerance for LL‐37 (Chernomordik et al., 2023) and that these cells have markers characteristic of immunologic memory (Chernomordik et al., 2020). Antibody generation is supported by T follicular (Tf) cells expressing the marker CXCR5 and persist as memory cells (Aloulou & Fazilleau, 2019; Crotty, 2011). They are found mainly in lymphoid organs but are also found in low quantities in circulation. CD4^+^ Tf helper cells provide functional support to B cells for antibody generation (Aloulou & Fazilleau, 2019; Crotty, 2011) although CD8^+^ Tf‐like cells modulate autoantibody responses (Chen et al., 2019; Elzein et al., 2021). It is unclear if Tf cells are potentially involved in the underlying mechanism of the autoantibody response to LL‐37 in an acute event.

LDL is a known binding partner of LL‐37 that stabilizes its presence in circulation (Sørensen et al., 1999) and alters LDL conformation such that it increases LDL uptake by macrophages (Nakamura et al., 2024). This interaction raises the question if the complexation between LL‐37 and LDL (LL‐37_LDL) will result in the generation of autoantibodies and if it does, whether these LL‐37_LDL IgG autoantibodies are reactive separately to either LDL or LL‐37 antigen alone, overlap with both antigens, or if the LL‐37_LDL complex generates a new antigenic source that has some overlap with LL‐37 and LDL antibodies. This may be important in the pathophysiology of ACS given that autoantibodies to LDL and its apolipoprotein component apoB‐100 are implicated in risk for coronary events (Björkbacka et al., 2016; Sjogren et al., 2008; Zhang et al., 2015).

In the current report, we first tested a cohort of subjects without ASCVD for intrinsic baseline LL‐37_LDL IgG immune reactivity as compared to LL‐37 IgG levels. We then evaluated LL‐37 IgG levels in samples of the AZACS study where ACS patients were randomized to a short course of azithromycin treatment or placebo and followed for 6 months for the occurrence of recurrent ischemic events or death (Cercek et al., 2003). Lastly, a cohort of patients hospitalized for ACS was also evaluated for LL‐37 IgG and Tf cell phenotype. The results suggest that although LDL intrinsically binds LL‐37 in circulation, LL‐37 IgG levels are higher compared to LL‐37_LDL IgG levels in healthy subjects. The results also show that LL‐37 IgG levels are elevated in subjects who had a prior MI and that ACS patients have LL‐37 reactive Tf cells further supporting the potential involvement of LL‐37 IgG in ACS.

## 2 MATERIALS AND METHODS

### 2.1 Patient samples

The investigation was approved by the Cedars‐Sinai Medical Center IRB and all studies were performed in accord with the Declaration of Helsinki.

Plasma samples (*N* = 6) were randomly selected from a subcohort of the EISNER (Early Identification of Subclinical Atherosclerosis by Noninvasive Imaging Research) Trial (NCT00927693) that had a coronary artery calcium scan (CACS) score of zero. The EISNER Trial was a community‐based cohort of asymptomatic subjects who underwent CAC scanning and cardiovascular risk assessment (Rozanski et al., 2011). Selected participants were middle‐aged with cardiovascular risk factors but no known prior coronary artery disease and gave written informed consent. The samples were selected for testing because of the verifiable absence of coronary artery disease at the time of sample collection. Additional information of the study design is detailed in Rozanski et al. (2011). Aliquoted samples were stored in −80°C until assays were performed.

The second cohort used for analysis were plasma samples (*N* = 500) collected for the AZACS study (Cercek et al., 2003). Briefly, the study was designed to evaluate the effect of short‐term treatment with azithromycin initiated after presentation with ACS on the occurrence of recurrent ischemic events and death during a 6 month follow up. Recruitment and baseline blood collection occurred between days 1 and 28 after admission prior to treatment randomization after obtaining written informed consent. Additional information of the study design is detailed in Cercek et al. (2003). Aliquoted samples were stored in −80°C until assays were performed. The current study was in compliance with Cedars‐Sinai Medical Center IRB approval (STUDY00003467). Patient characteristics are in Table 1. The study evaluated all available samples from the previously completed AZACS study to avoid selection bias.

**TABLE 1:** AZACS subcohort patient characteristics (*N* = 500).

Age	68 ± 13
Female	159 (32)
Male	341 (68)
Diastolic blood pressure (mm Hg)	67 ± 14
Systolic blood pressure	128 ± 22
Acute MI	264 (52.9)
History of hypertension	307 (61.5)
History of diabetes	142 (28.5)
History of raised lipids	312 (62.5)
History of smoking (active/ex)	234 (46.8)
Family history of heart disease	136 (27.2)
Previous MI	142 (28.4)
Hemoglobin (g/dL)	12.4 ± 6.1
WBC (×10^9^/L)	9.4 ± 9.7
Platelet (×10^9^/L)	188.5 ± 73
CK_MB (ng/mL)	9.4 ± 63.5
Troponin (ng/mL)	15.7 ± 44.7
Creatinine (mg/dL)	1.4 ± 4.8
Glucose (mg/dL)	130 ± 64.5
Total cholesterol (mg/dL)	180.5 ± 42.5
LDL cholesterol	106.3 ± 41.6
HDL cholesterol	43.1 ± 13.4
Triglyceride (mg/dL)	159.8 ± 138.2
Combined primary endpoint	60 (12)
Non‐fatal MI endpoint	12 (2.4)

*Note*: Categorical data presented as number of individuals (%); non‐categorical data presented as mean ± SD.

Abbreviations: CK‐MB, creatine kinase‐MB; HDL, high density lipoprotein; LDL, low density lipoprotein; MI, myocardial infarction; WBC, white blood cell count.

For the third cohort, plasma (*N* = 15) and PBMC (*N* = 11) samples were isolated from blood collected from patients with ACS (S‐T elevation and non S‐T elevation myocardial infarction) within 48 h of admission to the Cardiac Intensive Care Unit (CICU) at Cedars‐Sinai Medical Center to evaluate patients in the acute phase. Patients provided written informed consent under the approved Cedars‐Sinai Medical Center IRB protocol Pro00058160. Exclusions were inability to give informed consent, age less than 18 years old, active cancer treated with chemotherapy or radiation, patients taking immune‐suppressive drugs, and pregnant women. All subjects in the studies gave written informed consent for use of non‐identifiable data. Aliquoted plasma samples were stored in −80°C while cryopreserved viable PBMCs were stored under liquid nitrogen until assays were performed. Characteristics of ACS patients are in Table 2.

**TABLE 2:** Characteristics of ACS patients (*N* = 15).

Age	61.0 ± 10.1
Female	5 (33)
Male	10 (67)
Diabetes	3 (20)
Hypertension	8 (53)
Dyslipidemia	9 (60)
Past or current smoker	3 (20)
Statin use	6 (40)
PCI	15 (100)
WBC (×10^9^/L)	10.3 ± 3.2
Hemoglobin (g/dL)	14.3 ± 2.6
Platelet (×10^9^/L)	267.5 ± 53.7
Creatinine (mg/dL)	1.0 ± 0.2
BUN (mg/dL)	16.5 ± 4.2
Total cholesterol (mg/dL)	181.6 ± 59.8
LDL	114.9 ± 53.4
HDL	45.4 ± 11.1
Troponin peak	74.8 ± 68.3
LVEF % (echo)	50.7 ± 13.1
STEMI/NSTEMI	13/2

*Note*: Categorical data presented as number of individuals (%); non‐categorical data presented as mean ± SD.

Abbreviations: BUN, blood urea nitrogen; LVEF, left ventricular ejection fraction; PCI, percutaneous coronary intervention; STEMI/N, S‐T elevation myocardial infarction/non; WBC, white blood cell count.

### 2.2 Native (n)LDL IgG and LL‐37_LDL IgG

Commercially available plasma derived human native (n)LDL (Innovative Research; cat # IHULDL) and LL‐37 peptide (20 μg/mL each; Anaspec cat # AS‐61302) were combined (LL‐37_LDL) in PBS for 1 h in 37°C (Nakamura et al., 2024). After incubation, 100 μL of nLDL (20 μg/mL), LL‐37_LDL, or LL‐37 peptide were coated onto flat‐bottomed 96‐well polystyrene plates (MaxiSorp, Thermo Fisher; cat # 442404) overnight at 4°C. Plates were washed and blocked with 2% BSA (Sigma‐Aldrich; cat # A9418) in 1×PBS for 1 h in 37°C. Plates were washed and plasma samples diluted 1:100 in 1×PBS were added in duplicates and incubated for 1 h in 37°C. Plates were washed and HRP anti‐human IgG (Southern Biotech; cat # 2040‐05) was used as the detecting antibody with ABTS (Southern Biotech; cat # 0202‐01) as substrate color development. AB plasma pooled from healthy subjects (Innovative Research; cat # ISERAB100ML) was used as a calibrator for the assay and the values expressed as the ratio of each sample duplicate to the calibrator and averaged for the sample.

### 2.3 Anti‐LL‐37 IgG ELISA

Flat‐bottomed 96‐well polystyrene plates were pre‐coated with 100 μL LL‐37 (20 μg/mL) in Na_2_CO_3_‐NaHCO_3_ buffer (pH 9.6) overnight at 4°C to assess antibody levels using standard protocol. Each sample was assayed in duplicate. HRP anti‐human IgG was used as detecting antibody and color development with ABTS as substrate. Optical density values were recorded at 405 nm. Pooled AB plasma was used as calibrator for the assay and the values expressed as the ratio of each sample duplicate to the calibrator and averaged for the sample (Dimayuga et al., 2024). Intra‐assay CV was 6.8% while inter‐assay CV was 9.3%.

### 2.4 Modified activation induced marker (AIM) assay to detect Tf cells

To evaluate Tf cells in patient PBMCs, a modified AIM assay (Chernomordik et al., 2023; Chyu et al., 2005; Reiss et al., 2017) was used. Cryopreserved PBMCs were thawed, rinsed in anti‐aggregation solution (ImmunoSpot; cat #CTL‐AA‐010), and seeded in culture plates at a density of 3 × 10^6^ cells/mL in RPMI 1640 medium (Thermo Fisher; cat #11875093) supplemented with 10% heat‐inactivated pooled human serum (Innovative Research; cat #ISERABHI100ML) and 1× antibiotic/antimycotic (Gibco, Thermo Fisher cat # 15240062). After resting for 4 h, cells were preincubated with 0.5 μg/mL anti‐CD40 antibody (BD Bioscience; cat # 550391) for 15 min, then stimulated with 20 μg/mL LL‐37 whereas cells without treatment served as non‐stimulated control. Cells were harvested 18 h after seeding, stained for viability (LIVE/DEAD Fixable Aqua Dead Stain Kit, Thermo Fisher Scientific; cat # L34957), and subjected to cell surface staining for flow cytometry (BD LSRFortessa Cell Analyzer) using the following antibodies: CD4 (BD Bioscience; cat # 563550), CD8 (Invitrogen; cat # 48‐0088‐42), CD25 (BD Bioscience; cat # 555431), OX40 (CD134; BD Bioscience; cat # 563473), CD137 (4‐1 BB; Invitrogen; cat # 47‐1379‐42), CD154 (CD40L; Biolegend; cat # 310838) and CXCR5 (Biolegend; cat # 356903). Isotypes were used as staining control, and CD14 (cat 3 69‐0149‐42), CD16 (cat # 69‐0168‐42), and CD19 (cat # 69‐0168‐42) antibodies (Invitrogen) were used as dump staining to exclude B cells, DCs, macrophages, granulocytes, eosinophil cells, and neutrophil cells. Results are presented as percentage of gated CD4^+^ or CD8^+^ T cells.

### 2.5 Statistics

Continuous data presented as mean ± standard deviation (SD) or displayed as dot plots. D'Agostino & Pearson test was used to determine normal distribution. Mann–Whitney test was used to compare two unpaired groups with non‐normal distribution; Wilcoxon or Paired *T*‐test was used for paired comparison as appropriate and indicated in the Figure Legend. Correlation analysis was performed using Spearman test for non‐normal distribution or Pearson test for normal distribution. Comparison of the same samples with three different capture antigens for ELISA was performed using repeated measures ANOVA with Holm‐Sidak post‐hoc after confirming normal distribution by Kolmogorov–Smirnov test. *p* Value less than 0.05 was considered significant. Statistical analysis was performed using GraphPad Prism 10.0.0.

## 3 RESULTS

### 3.1 LL‐37 complexed to LDL binds IgG in healthy subjects

**FIGURE 1:**
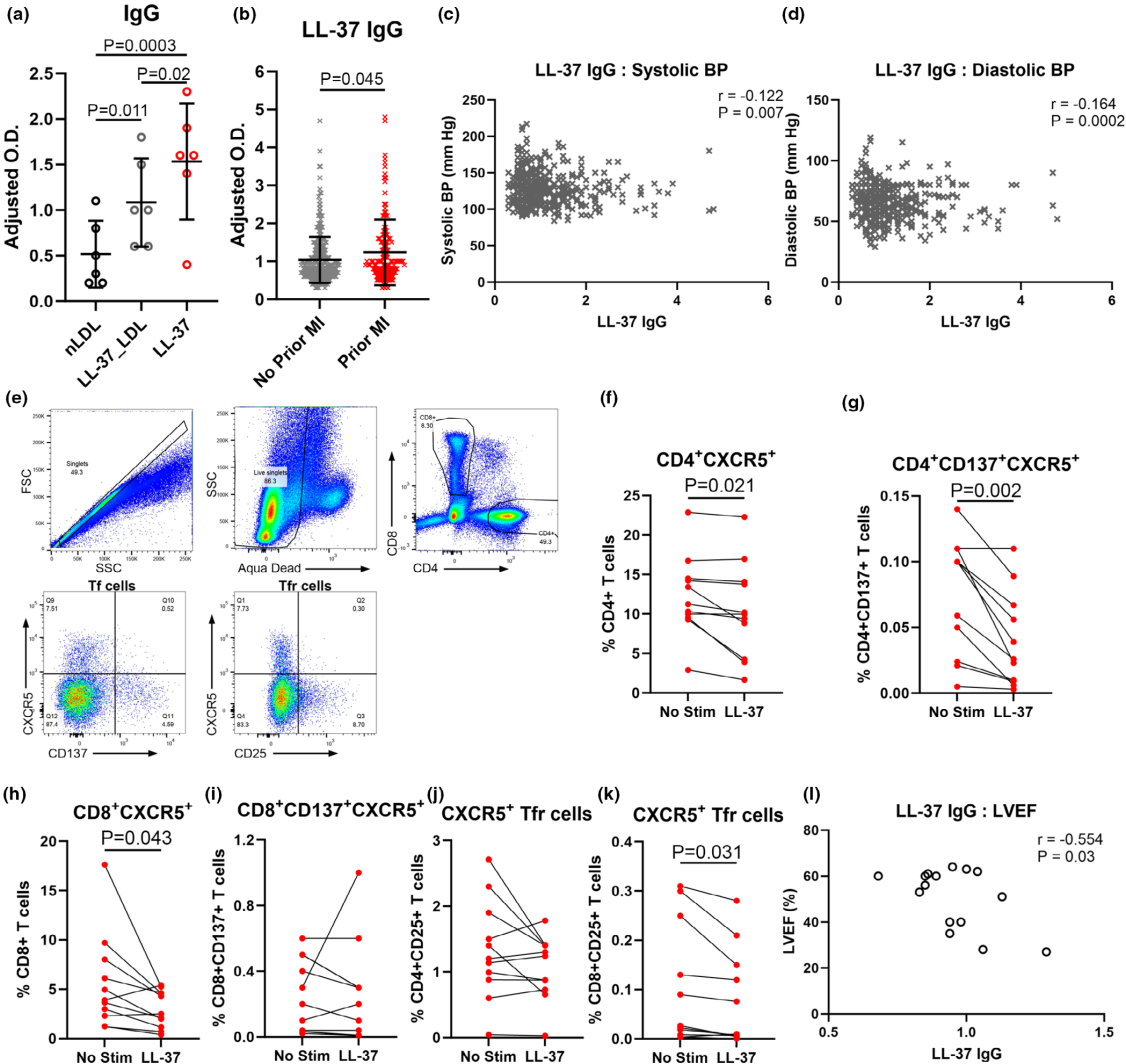
LL‐37 IgG levels and T follicular cell types in patients. (a) Autoantibodies against native LDL (nLDL), LL‐37‐LDL complex (LL‐37_LDL) and LL‐37 in plasma of healthy subjects with zero coronary artery calcium score (*N* = 6 subjects). Repeated measures one‐way ANOVA with Holm‐Sidak multiple comparison test. (b) LL‐37 IgG levels in AZACS patient plasma grouped according to occurrence or not of history of prior MI (*N* = 143 and *N* = 357 patients, respectively), Mann–Whitney test. LL‐37 IgG levels plotted against (c) systolic and (d) diastolic blood pressure; Spearman correlations. (e) Gating scheme for T follicular (Tf) and Tf regulatory (Tfr) cells. CD4+CXCR5+ Tf helper (f) and CD4+CD137+CXCR5+ Tf cells (g) cells in PBMCs of ACS patients stimulated with LL‐37. CD8+CXCR5+ Tf‐like (h) and CD8+CD137+CXCR5+ (i) Tf‐like cells in PBMCs of ACS patients stimulated with LL‐37 (*N* = 11). CD4+CD25+CXCR5+ (j) and CD8+CD25+CXCR5+ (k) Tfr cells in PBMCs of ACS patients stimulated with LL‐37 (*N* = 11), paired *T*‐test. (l) LL‐37 IgG levels plotted against LVEF in ACS patients (*N* = 15). Pearson correlation coefficient.

Reports have demonstrated that LL‐37 binds to (Sørensen et al., 1999) and modifies LDL structure such that macrophage uptake of LDL is enhanced (Nakamura et al., 2024). Given that both LDL and LL‐37 generate autoantibodies in atherosclerotic cardiovascular disease, we tested for the presence of autoantibodies reactive with the complexed molecules. To test this, plasma samples of healthy subjects with zero CACS randomly selected from the EISNER cohort (Rozanski et al., 2011) to clarify the steady‐state autoantibody levels in subjects without coronary artery disease. We evaluated the levels of IgG that are reactive with native (n)LDL, LL‐37 complexed with nLDL (LL‐37_LDL) (Nakamura et al., 2024) or LL‐37. There were significantly different IgG levels among the antigens tested, with nLDL reactive IgG having the lowest signal (95% CI: 0.133, 0.900) followed by LL‐37_LDL reactive IgG (95% CI: 0.576, 1.591) with approximately 2‐fold higher signal and LL‐37 reactive IgG (95% CI: 1.533, 2.203) with the highest signal at 3‐fold compared to nLDL (Figure 1a). The results are consistent with the report that LDL particles form complexes with LL‐37 (Nakamura et al., 2024; Sørensen et al., 1999) and provoke an autoantibody response.

### 3.2 LL‐37 IgG levels in patients with prior MI in the AZACS cohort

Our prior report of increased levels of LL‐37 IgG in baseline plasma of subjects that had an MI during a 13‐year follow up (Dimayuga et al., 2024) suggested that the autoantibody level may be elevated early in the process that leads to MI. Extending this notion, it also suggests that LL‐37 IgG levels may be different between ACS patients who had a history of prior MI compared to those who did not. Patients that had a prior MI had a modest but significant 20% increase in LL‐37 IgG levels (95% CI: 0.97, 1.10) compared to patients with no prior MI (95% CI: 1.09, 1.38; Figure 1b). This suggests that not only is LL‐37 IgG elevated in subjects pre‐disposed to an MI event in the future (Dimayuga et al., 2024), but that this persists even after a prior MI.

### 3.3 LL‐37 IgG level correlates with blood pressure in AZACS cohort

Given the reported associations between plasma levels of LL‐37 and blood pressure (Höpfinger et al., 2024), we also evaluated the relationship of LL‐37 IgG with clinical data. LL‐37 IgG levels were negatively correlated with systolic (Figure 1c) and diastolic (Figure 1d) blood pressure. Statistical significance remained after adjusting for medication (diuretics and ace inhibitors) and creatinine level (SBP adjusted *p* = 0.0169; DBP adjusted *p* = 0.0167). The results are relevant to the previously reported association of LL‐37 levels with blood pressure (Höpfinger et al., 2024).

### 3.4 Tf cell response to LL‐37 in the ACS group

Antibody generation and production is supported by Tf cells (Aloulou & Fazilleau, 2019). The AZACS study was limited to collecting plasma samples and did not include cryo‐preservation of viable PBMCs. We thus characterized the T follicular cell response to LL‐37 in PBMCs from an ACS cohort collected within 48 h of admission to the CICU. Tf helper (Tfh) cells are classified as CXCR5^+^ (Aloulou & Fazilleau, 2019) while CD137 expression marks T cell activation and is crucial in generating Tf memory response (Alfaro et al., 2015; Oh et al., 2015). ACS PBMCS were either unstimulated or stimulated with LL‐37 and the cells analyzed according to the gating depicted in (Figure 1e). LL‐37 stimulation resulted in significantly reduced CD4^+^CXCR5^+^ (Figure 1f) and CD4^+^CD137^+^CXCR5^+^ (Figure 1g) Tfh cells. A similar CD8^+^ follicular‐like T cell type that is CXCR5^+^ (Chen et al., 2019; Elzein et al., 2021) was also significantly reduced (Figure 1h). On the other hand, no reduction in CD8^+^CD137^+^CXCR5^+^ Tf‐like cells were observed after LL‐37 stimulation (Figure 1i). The results suggest that CD8^+^CD137^+^CXCR5^+^ Tf‐like cells may be mechanistically involved in supporting LL‐37 autoantibody generation and persistence in acute coronary events. One other potential mechanism for persistent antibody levels are reduced levels of T follicular regulatory (Tfr) cells, which are considered follicular cells in phenotype with the added expression of CD25 which functions as T regulatory cells specific to Tf response (Aloulou & Fazilleau, 2019). There was no significant effect on CD4^+^CD25^+^CXCR5^+^ Tfr cells (Figure 1j) yet a significant reduction in CD8^+^CD25^+^CXCR5^+^ Tfr cells (Figure 1k) observed after LL‐37 stimulation of ACS PBMCs. Thus, the constant presence CD8^+^CD137^+^CXCR5^+^ Tf‐like cells in response to LL‐37 that occurred even as other Tf cell types were reduced in the context of reduced CD8^+^CD25^+^CXCR5^+^ Tfr cells suggest a lack of CD8^+^CD137^+^CXCR5^+^ Tf‐like cell regulation.

### 3.5 LL‐37 IgG levels are correlated with LVEF in the ACS group

A previous report demonstrated that LL‐37 levels were negatively correlated with LVEF (Höpfinger et al., 2024). The AZACS data collected did not include LVEF and therefore could not be assessed. We therefore evaluated the relationship between LL‐37 IgG levels and LVEF in a cohort of ACS patient samples collected within 48 h of admission to the CICU. There was a negative correlation between LL‐37 IgG levels and LVEF in ACS patients (Figure 1l). The results are consistent with the prior report on the association between cardiac function and LL‐37 levels (Höpfinger et al., 2024) and further support the involvement of LL‐37 IgG in the acute event (Dimayuga et al., 2024).

## 4 DISCUSSION

LL‐37 is known to bind LDL through interaction with apoB‐100 (Sørensen et al., 1999). Our results demonstrate that autoreactive antibodies bind to LL‐37_LDL complex but that LL‐37 IgG levels are comparably higher. More detailed and specific tests need to be performed to further characterize this. However, the results suggest that LL‐37 IgG‐ICs (Dimayuga et al., 2024) have LDL, or fragments thereof, bound to these complexes. The results also suggest that LL‐37 immune reactivity is intrinsically evident and not merely a bystander to apoB‐100 immune responses. Given that anti‐LDL and anti‐apoB‐100 autoantibodies are implicated in CAD and considering their known interaction with LL‐37, it is possible that there is a shared step in the process that generates autoimmune responses among these autoantigens.

We previously reported increased LL‐37 IgG levels in subjects predisposed to have a future MI and its potential mechanistic involvement in immunothrombosis was mediated in part by LL‐37 IgG‐IC activation of platelets through FcɣRIIa (Dimayuga et al., 2024). In this report, we demonstrated that LL‐37 IgG levels are elevated in ACS patients who had a prior MI, consistent with the finding that higher relative levels of LL‐37 IgG are potentially involved in the immunothrombotic process in MI (Dimayuga et al., 2024). However, given the difference in sample collection time and stage of disease in the current patient cohort compared to the prior report (Dimayuga et al., 2024), it is also possible that higher LL‐37 IgG levels may be due to the prior MI itself and/or broader immune activation.

The modest negative association of LL‐37 IgG levels with blood pressure may be relevant to prior reports of a positive association between LL‐37 levels (Höpfinger et al., 2024) or LL‐37 mRNA expression by PBMCs (Benachour et al., 2009) and blood pressure. A positive association of LL‐37 level with blood pressure suggests, by extension, that a process wherein LL‐37 bound to endogenous LL‐37 autoantibodies in circulation may mask its detection by standard antibody capture‐based ELISA. Based on these findings, we speculate that patients with lower antibody levels have less binding to and sequestration of LL‐37 into IgG immune complexes and would have a higher amount of free LL‐37 detected in circulation, as reported in the work by Höpfinger et al. (2024) and thus LL‐37 levels in their report and LL‐37 IgG levels in the current report reciprocate each other. The more modest association of LL‐37 IgG with blood pressure as compared to the stronger association with LL‐37 peptide levels in the other reports (Benachour et al., 2009; Höpfinger et al., 2024) is potentially due to the nature of the assays used. Our LL‐37 IgG assay is an indirect, relative measure (expressed as optical density relative to pooled healthy samples) as compared to the direct peptide amount measured in other reports (Höpfinger et al., 2024). The significance of these associations is highlighted by reports on the role of the immune system in hypertension (Harrison & Patrick, 2024). The potential role of LL‐37 as a constituent of NETs is relevant in this context (Bosch, 2011; Lande et al., 2011). Increased plasma levels of NETs were reported in patients with newly diagnosed essential hypertension, which were subsequently reduced by angiotensin II receptor blocker treatment (Chrysanthopoulou et al., 2021). Mechanistic studies further provided evidence on the role of NETs (Krishnan et al., 2024). However, the potential role of LL‐37 IgG in hypertensive vascular disease remains unclear.

Antibody production and class switching by B cells are mediated in part by Tf cells which are found mainly in lymphoid organs (Aloulou & Fazilleau, 2019; Crotty, 2011). However, Tf cells are also detected in circulation in very low numbers. In our study, CXCR5^+^ Tf cells in PBMCs were generally reduced after LL‐37 stimulation except for CD8^+^CD137^+^CXCR5^+^ Tf‐like cells suggesting that this may be the Tf cell population that supports LL‐37 IgG levels, but functional studies need to be performed to confirm this. The role of CD8^+^CXCR5^+^ Tf‐like cells in shaping autoantibody responses has been demonstrated both in humans and mice (Chen et al., 2019; Elzein et al., 2021). One possible mechanism that is permissive to Tf cell presence is the reduced level of Tfr cells that regulate antigen‐specific response and function of CD8^+^CD137^+^CXCR5^+^ Tf‐like cells, such as supporting autoantibody production (Chen et al., 2019; Elzein et al., 2021). Reduced CD8^+^CD25^+^CXCR5^+^ Tfr cells in response to LL‐37 stimulation could lead to unchecked LL‐37 IgG generation (Aloulou & Fazilleau, 2019; Chen et al., 2019; Crotty, 2011; Elzein et al., 2021) but functional studies need to be performed.

The negative association between LL‐37 IgG and LVEF in the acute patients suggests the potential role of LL‐37 in heart function, relevant to a prior report by Höpfinger et al. demonstrating a negative correlation between LL‐37 levels and LVEF in a recurrent ACS subcohort (Höpfinger et al., 2024). The acute event in ACS results in an abrupt neutrophil activation and discharge of NETs (Mangold et al., 2015) that increases LL‐37 in the study by Höpfinger et al. The negative association between LL‐37 IgG and LVEF in the current study may reflect the chronic state of IgG levels at the time of the acute event in ACS subjects. The reported elevated LL‐37 IgG levels in otherwise healthy subjects who have a future MI is consistent with a chronic condition (Dimayuga et al., 2024). The potential significance of our findings is further highlighted by the reported negative association between NETs burden and infarct size in STEMI patients (Mangold et al., 2015), that was subsequently confirmed by the negative association between extracellular double stranded DNA levels in blood samples at the culprit lesion site and LVEF (Mangold et al., 2022). LL‐37 binds extracellular DNA (Zhang et al., 2010) and both are components of NETs (Villanueva et al., 2011) which are structurally involved in thrombus formation (Mangold et al., 2015). Proteomic studies have demonstrated the presence of both LL‐37 and IgG in coronary and carotid thrombi proteomes (Alonso‐Orgaz et al., 2014; Muñoz et al., 2018). Combined with our prior report on the role of LL‐37 IgG in promoting platelet activation (Dimayuga et al., 2024), the results suggest that LL‐37 IgG may be detrimental in ACS.

The use of three separate cohorts in the investigation was necessary to answer specific questions. The healthy cohort was used to establish the basal immune reactivity of LL‐37 itself relative to its binding complex with LDL (Sørensen et al., 1999; Zhang et al., 2015) that is known to occur. The use of the AZACS cohort provided a larger sample size to evaluate its potential pathophysiologic role in ACS, confirming a previous report (Dimayuga et al., 2024) and with sufficient power to evaluate clinical associations such as blood pressure. The last cohort was necessary to evaluate the T cell response relevant to persistent LL‐37 IgG levels in ACS. The combined results of the 3 different cohorts provide separate but linked evidence supporting the role of LL‐37 IgG in the pathophysiology of ACS.

Limitations of the study include potential bias introduced by the retrospective use of samples from the previous AZACS study (Cercek et al., 2003) and the small number of the ACS patient cohort from a single center that cannot account for other factors. Validation of the studies in a larger patient cohort including stable CAD patients is needed to confirm the results. Another limitation is the lack of more extensive mechanistic studies, hampered by the differences in the nature and function of LL‐37 and the mouse counterpart CRAMP, making these studies challenging (Dimayuga et al., 2024; Nakamura et al., 2024).

In conclusion, our study suggests that ACS patients who had a prior MI have significantly higher LL‐37 IgG levels. The association between blood pressure and LL‐37 IgG levels in the AZACS cohort is relevant to another report (Höpfinger et al., 2024) and in addition to the negative correlation with LVEF suggests a potential role in ACS. CD8^+^CD137^+^CXCR5^+^ Tf‐like cells in response to LL‐37 coupled with reduced Tfr cells may be relevant to the elevated levels of LL‐37 IgG in at‐risk patients. Combined with our previous report suggesting that LL‐37 IgG response propagates immunothrombosis in MI (Dimayuga et al., 2024), the results support a potential role for LL‐37 IgG in ACS.

## AUTHOR CONTRIBUTIONS

**Paul C. Dimayuga:** Conceptualization; data curation; investigation; methodology; project administration; validation. **Jianchang Zhou:** Formal analysis; investigation; methodology; validation. **Xiaoning Zhao:** Formal analysis; investigation; methodology; validation. **Kuang‐Yuh Chyu:** Conceptualization; formal analysis; investigation; methodology. **Prediman K. Shah:** Conceptualization; funding acquisition; resources; supervision. **Bojan Cercek:** Conceptualization; data curation; funding acquisition; project administration; supervision.

## FUNDING INFORMATION

This work was supported by The Heart Foundation; Eisner Foundation; Peterson Foundation; Corday Foundation; Spielberg Fund (to PKS); and The Eleanor and Harold Foonberg Endowed Chair in Cardiac Intensive Care Fund (to BC).

## CONFLICT OF INTEREST STATEMENT

No conflicts of interest.

## ETHICS STATEMENT

The investigation was approved by the Cedars‐Sinai Medical Center IRB and all studies were performed in accord with the Declaration of Helsinki.

## Data Availability

The data that support the findings of this study are available from the corresponding author upon reasonable request.
